# Current Strategies and Future Directions to Achieve Deep Molecular Response and Treatment-Free Remission in Chronic Myeloid Leukemia

**DOI:** 10.3389/fonc.2020.00883

**Published:** 2020-06-02

**Authors:** Mario Annunziata, Massimiliano Bonifacio, Massimo Breccia, Fausto Castagnetti, Antonella Gozzini, Alessandra Iurlo, Patrizia Pregno, Fabio Stagno, Giorgina Specchia

**Affiliations:** ^1^Hematology Division, AORN Cardarelli-Napoli, Naples, Italy; ^2^Section of Hematology, Department of Medicine, University of Verona, Verona, Italy; ^3^Department of Translational and Precision Medicine, Sapienza University, Rome, Italy; ^4^Department of Experimental, Diagnostic and Specialty Medicine, Institute of Hematology “L. and A. Seràgnoli”, “S. Orsola-Malpighi” Univeristy Hospital, University of Bologna, Bologna, Italy; ^5^Department of Cellular Therapy and Transfusional Medicine, AUO Careggi, Florence, Italy; ^6^Hematology Division, Foundation IRCCS Ca' Granda Ospedale Maggiore Policlinico, Milan, Italy; ^7^Hematology Division, Oncology and Hematology Department, AOU Città della Salute e della Scienza di Torino, Turin, Italy; ^8^Division of Hematology and Bone Marrow Transplant, AOU Policlinico—V. Emanuele, Catania, Italy; ^9^Hematology Section, Department of Emergency and Organ Transplantation, University of Bari-Aldo Moro, Bari, Italy

**Keywords:** chronic myeloid leukemia, deep molecular response, optimal strategies, treatment-free remission, tyrosine kinase inhibitors

## Abstract

The treatment of chronic myeloid leukemia (CML) has been radically changed by the approval of tyrosine kinase inhibitors (TKIs), which target BCR-ABL1 kinase activity. CML is now managed as a chronic disease requiring long-term treatment and close molecular monitoring. It has been shown that in a substantial number of patients who have achieved a stable deep molecular response (DMR), TKI treatment can be safely discontinued without loss of response. Therefore, treatment-free remission (TFR), through the achievement of a DMR, is increasingly regarded as a feasible treatment goal in many CML patients. However, only nilotinib has approval in this setting and a number of controversial aspects remain regarding treatment choices and timings, predictive factors, patient communication, and optimal strategies to achieve successful TFR. This narrative review aims to provide a comprehensive overview on how to optimize the path to DMR and TFR in patients with CML, and discusses recent data and future directions.

## Introduction

The approval of tyrosine kinase inhibitors (TKIs), which target BCR-ABL1 kinase activity, has significantly reduced the mortality rate associated with chronic myeloid leukemia (CML) and revolutionized treatment (1). CML is now managed as a chronic disease requiring long-term treatment. Currently, five TKIs are approved for the treatment of CML: imatinib, the first TKI approved for this indication; second-generation TKIs, nilotinib, dasatinib, and bosutinib; and ponatinib, a third-generation TKI (2–6). Imatinib, nilotinib, and dasatinib are currently recommended for both first- and second-line treatments, bosutinib and ponatinib for second and later lines (7, 8). Compared with imatinib, newer TKIs are more potent, act more rapidly, and are associated with higher response rates (9–11).

The European LeukemiaNet (ELN) and European Society for Medical Oncology (ESMO) guidelines recommend basing the choice of first-line TKI on treatment goal, age and comorbidities, considering the safety profile of the TKI (7, 8). Indeed, distinct safety profiles for each of imatinib, nilotinib, and dasatinib have emerged from clinical trials and clinical practice (9, 12–14).

Evidence from several studies suggests that in patients who have achieved a sustained stable, deep molecular response (DMR), TKI treatment can be safely discontinued with close monitoring without relapse, despite BCR-ABL DNA remaining detectable (15–22). As a consequence, DMR yielding treatment-free remission (TFR) is increasingly regarded as a feasible treatment goal in CML (23), however, TFR is only successful in 40–60% of patients (24).

With the growing availability of clinical trials and real-life data on TFR in recent years, it has begun to seem increasingly feasible to offer to CML patients a safe, informed, and shorter path to TFR, through achieving a DMR. However, many controversial aspects remain regarding treatment choices and timings, predictive factors, patient communication, and optimal strategies to achieve successful TFR.

The aim of this review is to provide an update on CML management to achieve TFR, and to discuss recent data and future directions.

## Who Is a Candidate for Treatment-free Remission Today

For the CML scientific community, TFR represents a striking result of the last decade. However, two crucial questions remain uppermost in the CML research agenda: (1) Can we further extend the TFR opportunity to more patients? (2) How can we accurately predict successful TFR or failure, in order to optimize the patient-specific treatment strategy and duration?

### Guidelines vs. the Real-Life Treatment-Free Remission Population

The 2013 ELN recommendations state that CML patients with optimal treatment response should continue treatment indefinitely, with the possible consideration of TKI discontinuation in individual patients only if a certified high-quality monitoring at monthly intervals is available, or in the context of well-designed, prospective, controlled studies (7). While the newly published 2020 ELN recommendations (25) mention TFR as a new significant goal in CML, they do not provide a guide in treatment optimization to achieve TFR with the current drugs available. Indeed, the Italian GIMEMA CML Working Party (26) discuss and suggest treatment policies to maximize TFR achievement, considered virtually a feasible goal for all patients.

To date more than 30 clinical trials on TFR have been conducted, and discontinuation of TKI therapy has been shown to be feasible in patients with durable DMR (8, 27, 28), with ~50% of patients reaching a successful discontinuation with imatinib or second-generation TKIs (dasatinib and nilotinib). In addition, criteria for stopping TKIs outside of clinical trials (Table 1) have been suggested based on expert recommendations (24, 30), and formal guidelines [ESMO (8) and the National Comprehensive Cancer Network (NCCN) (29)].

**Table 1:** A summary of criteria for tyrosine kinase inhibitor discontinuation in clinical practice.

**Criteria**	**ELN 2020 (25)^a^**	**NCCN 2018 (29)^a^**	**ESMO 2017 (8)^a^**	**Hughes et al. (24)^b^**
Age		≥ 18 yr		
Institutional criteria for safe supervision^*^	Yes		Yes	Yes
History of CML	CP only	CP only	CP only	CP only
Sokal score at diagnosis			Non-high	Non-high
*BCR-ABL* transcript	Minimal criteria: Typical (e13a2 or e14a2)	Quantifiable	Typical	Typical (e13a2 or e14a2)
Response to first-line TKI	Minimal criteria: No prior treatment failure (except intolerance of first-line TKI)	No resistance	Optimal	Optimal
Duration of TKI therapy	Minimal criteria: > 5 yr (> 4 yr for 2GTKI) Optimal criteria: >5 yr	≥ 3 yr	≥ 5 yr	> 8 yr
Depth of DMR	MR^4^	MR^4^	MR^4.5^	MR^4.5^
Duration of DMR	Minimal criteria: ≥ MR^4^ for >2 yr Optimal criteria: >3 yr if MR^4^ or >2 yr if MR^4.5^	≥2 yr	≥MR^4^ for ≥2 yr	>2 yr
Frequency of PCR monitoring	Monthly for 6 mo, every 2 mo for 6 mo, every 3 mo thereafter	Monthly for 1 yr, every 6 wks for 1 yr, every 12 wks thereafter	Monthly for 6 mo, every 6 wks for 6 mo, every 3 mo thereafter	Every 4–6 wks
PCR sensitivity		At least MR^4.5^		
PCR turnaround time		Within 2 wks		Within 4 wks
Resumption of TKI		Within 4 wks of MMR loss		

*2GTKI, second-generation TKI; CML, chronic myeloid leukemia; CP, chronic phase; DMR, deep molecular response; ELN, European LeukemiaNet; ESMO, European Society for Medical Oncology; MR, molecular response; MMR, major molecular response; mo, month; NCCN, National Comprehensive Cancer Network; PCR, polymerase chain reaction; RQ-PCR, real-time quantitative reverse transcriptase-PCR; TKI, tyrosine kinase inhibitors; wks, weeks; yr, years*.

* *High-quality, internationally standardized, accurate, sensitive RQ-PCR laboratory; RQ-PCR test results within 4 weeks; Ability to run RQ-PCR testing every 4–6 weeks, if necessary; Follow-up strategy in place to enable rapid treatment-resumption for loss of MMR*.

a *Guidelines*.

b *Expert opinion*.

According to the ESMO Clinical Practice Guidelines, the availability of appropriate, high-quality and certified monitoring is imperative when considering treatment discontinuation in patients outside of clinical trials (8). Prerequisites for safe stopping include: institutional requirements for safe supervision, the identification of typical *BCR-ABL1* transcripts at diagnosis, at least 5 years of TKI therapy, achievement of MR^4.5^, and a stable DMR (at least MR^4^) for at least 2 years (Table 1). Notably, longer TKI therapy and longer DMR improve the stability of TFR (31).

The NCCN guidelines (Version 1.2019) for CML state that, outside of a clinical trial, discontinuation of TKI therapy appears to be safe in selected CML patients (29, 32). Criteria for TKI discontinuation include: age (≥18 years), chronic phase CML (CP-CML; no prior history of accelerated or blast phase), prior TKI therapy for ≥3 years, stable DMR (defined as at least MR^4^; *BCR-ABL1* ≤0.01% IS) for ≥2 years, and unceasing molecular monitoring (Table 1). The new 2020 ELN recommendations, recently published, also define minimal and optimal criteria for discontinuation (Table 1). Thus, available recommendations are discordant at some points, and, despite numerous clinical trials demonstrating TFR feasibility in CML patients with DMR, the optimal selection criteria for patients in clinical practice remain uncertain (33), especially regarding the definition of DMR depth and its duration, which seem to be the most important variables associated with a successful TFR.

A number of studies have investigated clinical and biologic predictive factors related to TFR success, aiming to delineate the best criteria for discontinuation eligibility, however to date these factors are far from impacting clinical choices, and a validated TFR predictive model has not yet been developed.

In the imatinib suspension and validation (ISAV) study, both age (<45 years) and a positive digital PCR status were shown to predict relapse in CML patients who discontinued treatment with imatinib (34). Indeed the highest risk of relapse was identified in younger patients (<45 years) with digital PCR positivity (100% at 15 months), suggesting that discontinuation of imatinib was better achievable in the context of a continual negative digital PCR status.

In IMMUNOSTIM, a translational study within the STIM trial, significantly higher numbers of natural killer (NK) cells were identified in non-relapsing compared with relapsing CP-CML patients in DMR after imatinib discontinuation, suggesting that NK cells may be associated with molecular relapse-free survival (MRFS) after cessation of imatinib (35).

The EURO-SKI also identified the importance of NK cells in maintaining remission after imatinib discontinuation in CML patients, suggesting an association between an increase in MRFS and a higher proportion of NK cells, which were mature and secreted TNF-α/interferon (IFN)-γ cytokine in non-relapsing patients (36).

A significant positive correlation between the frequencies of innate CD8(+) T-cells and NK cells was identified in CML patients with TFR for ≥2 years after TKI discontinuation, which is suggestive of a link between these lymphocyte populations and may represent a novel innate biomarker of successful TFR (37).

Younger age at discontinuation and line of treatment were the only predictive factors that affected TFR in a univariate analysis but these lost significance in the subsequent multivariate analysis performed retrospectively in 293 CP-CML Italian patients in DMR who discontinued TKI therapy (38). The DASFREE study reported older age as a favorable prognostic factor of 2-years TFR in patients with CML (age >65 years; *p* = 0.0012) (39). Given these results, current recommendations do not consider age as a limiting factor for discontinuation eligibility (26).

The potential predictive role of *BCR-ABL* transcripts (e13a2 and e14a2) in achieving a sustained DMR and TFR was assessed in a number of studies, as recently reviewed (40). Indeed, several large studies have shown transcript type to be associated with outcome (41) or not associated with survival outcome (42, 43). Even if an association with outcome is controversial, a higher incidence of sustained DMR and TFR success was shown for patients harboring the e14a2 transcript vs. e13a2, possibly due to differences in immunogenicity, technical or genetic reasons (41).

In patients with atypical *BCR-ABL1* transcripts, TKI discontinuation is not recommended in current guidelines unless standardized methods for response monitoring are available (see Table 1). However, TKI discontinuation was recently reported in case series of seven patients carrying atypical transcripts, with six out of seven patients remaining off-treatment after a median follow-up of 25 months (range 5–77 months) (44). Further results are needed in this setting to confirm these data.

As additional biologic criteria, the potential role of residual leukemic stem cells (LSC) on TFR failure has been recently investigated. Interestingly, the results have shown that flow cytometry of peripheral blood could also detect residual circulating CD26^+^ LSC in stable TFR patients, even if with fluctuating values, with a progressive increase in relapsed patients (45). Notably, this study showed no difference in CD26+ peripheral blood LSC between patients who relapsed or remained in remission after TKI discontinuation.

Besides LSC, the lineage of residual BCR-ABL1-positive cells is also under evaluation. A recent study from 20 CML patients in TFR showed that leukemic lymphocytes appeared to be long lived and may contribute to measurable residual disease (46).

Even if many factors are investigated, the “optimal” conditions for TFR require further validation. Current recommendations are in support of initial proposal at baseline of TFR strategy, informing on the minimum conditions for TFR eligibility, among which, the achievement of a stable DMR is seen as the most critical factor.

## Optimizing Treatment to Reach a Deep Molecular Response

The availability of multiple treatment options is a major advance in CML management, but selecting the most appropriate TKI can be challenging in routine clinical practice, and several practical issues are still poorly defined by current guidelines.

### First-Line Treatments: Clinical Trials and Real-Life Data

The choice of first line therapy should take into account tolerability, safety, age, and comorbidities, which may predict specific toxicities with different TKIs (7), as shown by phase III pivotal trials in large populations of newly diagnosed, treatment-naïve CML patients (9, 10, 12, 47). The ENESTnd trial, demonstrated a better long-term efficacy of nilotinib compared with imatinib, that was recently confirmed in the 10-years follow-up analysis (12, 48). By 5 years, significantly more patients treated with nilotinib either 300 or 400 mg twice daily achieved a major molecular response (MMR) (77.0 and 77.2 vs. 60.4%, respectively, *p* < 0.0001 for both comparisons vs. imatinib) and DMR (65.6 and 63.0 vs. 41.7% for MR^4^, and 53.5 and 52.3 vs. 31.4% for MR^4.5^, respectively; *p* < 0.0001 for all comparisons vs. imatinib) (12). At 10 years follow up, cumulative MMR rates were 82.6 and 80.4 vs. 69.6%, cumulative MR^4^ rates were 72.7 and 68.7 vs. 55.5%, and cumulative MR^4.5^ rates were 63.8 and 61.6 vs. 45.2%, respectively, and both nilotinib arms were associated with a significantly lower probability of CML transformation from the chronic phase to the accelerated and blast phases (48).

The 5-years results of the DASISION trial similarly showed that dasatinib 100 mg once daily was able to induce deeper and faster molecular response compared with imatinib 400 mg once daily (MMR 76 vs. 64%, *p* = 0.0022; MR^4.5^ 42 vs. 33%, *p* = 0.0251) (9). Differently from the ENESTnd trial, the number of progressions was not significantly inferior in patients receiving dasatinib.

Published evidence also suggests that first-line treatment with second-generation TKIs is successful in elderly CML patients (49). A sub-analysis of the open-label ENEST1st trial, which evaluated first-line nilotinib 300 mg twice daily in over 1,000 CML patients, suggested that age did not influence response rates and that DMR can also be achieved in elderly patients (49). After 18 months of treatment with nilotinib, the rate of MR^4^ in the overall population was 38.4%; age-subgroup analyses revealed similar MR^4^ rates in patients aged 18–39, 40–59, 60–74, and ≥75 years (33.9, 39.6, 40.5, and 35.4%, respectively). The MR^4.5^ rates at 18 months were also similar across age-subgroups (18.0, 22.4, 21.8, and 14.6%, respectively); the incidence of adverse cardiovascular events, including arterial thrombotic events, was significantly different in the age classes, suggesting caution in elderly patients aged ≥60 years.

In a real-life quantification of CP-CML patients treated with first-line imatinib, dasatinib, or nilotinib (320, 24, and 53 patients, respectively, with a median follow-up of 9 years for patients treated with imatinib, and 3 years for dasatinib- and nilotinib-treated patients), 13, 29, and 45% of patients, respectively, were eligible for TFR after a median TKI treatment of 3 years (50). These data, which are in line with previous clinical trials results, suggest that a sustained DMR may be induced by second-generation TKIs over a shorter median time compared with imatinib, and may enable an early TFR eligibility. Thus, an appropriate selection of patients at baseline can increase the proportion of potential candidates eligible for TKI discontinuation.

### Second-Line Treatments: Early and Late Switch

Current recommendations do not provide a usable algorithm aimed to delineate the best strategy of achieving DMR, either through early or late switch, for patients whose response is not early or not deep.

In a retrospective Chinese study, higher DMR rates have been reported following early switching to nilotinib in CP-CML patients with a warning molecular response at 3 months compared with patients who continued imatinib treatment (51). In this study, 46 patients had a warning response at 3 months defined according to ELN-2013 recommendations, 26 patients continued with imatinib, and 20 patients switched to nilotinib 300 or 400 mg twice daily. With the limitation of the absence of randomization, a higher percentage of patients receiving nilotinib achieved a MR^4^ (61.5 vs. 18.6%; *p* = 0.035) by 4 years, compared with imatinib-treated patients; however, a higher proportion of high Sokal score patients was observed in patients receiving imatinib and the number of treatment interruptions was not reported.

Regarding late switch, the prospective ENEST-Complete Molecular Remission (ENEST-cmr) trial reported that, in 207 CML patients with stable cytogenetic response but detectable *BCR-ABL1* levels on long-term imatinib, switching to nilotinib was more effective for achieving DMR than continuing with imatinib (52). Patients were randomized between switching to nilotinib 400 mg twice daily, or continuing imatinib 400 mg once daily. By 24 months, the rates of MR^4.5^ were 42.9 and 20.8%, respectively among patients without MR^4.5^ at study start; among patients without MMR at study start, or among patients in MMR but without MR^4.5^ at study start, a MR^4.5^ by 2 years was achieved by 29.2 vs. 3.6% and 47.3 vs. 27.9% of patients in the nilotinib and imatinib arms, respectively. The final 48-months ENEST-cmr results (53) confirm that continued imatinib therapy is associated with lower DMR rates and also suggests that a late switch (after 2 years from enrollment) is less effective.

### Treatment-Free Remission

The long-term durability and safety of TFR following frontline nilotinib treatment were demonstrated in the 192-weeks results of the ENESTfreedom study (54). ENESTfreedom was a single-arm, phase 2 trial which assessed the potential for TFR in patients with CP-CML who had ≥2 years of frontline nilotinib treatment and sustained DMR during a 52-weeks nilotinib consolidation phase (defined as MR^4.5^ in the last assessment, no assessment worse than MR^4^, and ≤2 assessments between MR^4^ and MR^4.5^) (16). In total, 215 patients entered the 52-weeks nilotinib consolidation phase and 190 patients (88.4%) with sustained DMR throughout were eligible for the TFR phase. In the TFR population, the median duration of nilotinib prior to TFR was 43.5 months, and 98 patients (51.6%; 95% CI, 44.2–58.9%) remained in remission after 1 year. At 192 weeks, the TFR rate was 44.2% (84 of 190 patients), MMR and MR^4.5^ were regained by 98.9% (90 of 91) and 92.3% (84 of 91) of patients, respectively, who resumed nilotinib (54). There were no disease progressions or CML-related deaths, and musculoskeletal pain-related events were progressively reduced from the first to the 4th year of discontinuation.

Likewise, the long-term durability and safety of TFR following second-line nilotinib was demonstrated in the 192-weeks results of the ENESTop study (55). In this study, patients were eligible to attempt TFR after ≥3 years of nilotinib treatment and without confirmed loss of MR^4.5^ after a 1 year consolidation phase. Of the 126 CP-CML patients with sustained DMR on second-line nilotinib who entered the TFR phase, 56 patients completed ≥192 weeks of TFR, 59 patients resumed nilotinib, and 11 patients discontinued the study. The TFR rate at 192 weeks was 46.0% (58 of 126 patients), with 57 patients in MR^4.5^. MR^4^ and MR^4.5^ were regained by 94.9% (56 of 59) and by 93.2% (55 of 59) of patients, respectively, who resumed nilotinib. Overall, there were no deaths related to CML and no cases of disease progression reported.

Based upon these two studies, nilotinib has received Health Authority approval for attempting TFR.

In the 2-years update of the DASFREE study, including 84 patients with CP-CML and sustained DMR, TFR at 2 years following dasatinib discontinuation was 46% (39). In this study, patients were eligible to attempt TFR if they had received ≥2 years of dasatinib treatment and had dasatinib-induced MR^4.5^ for ≥1 year prior to study entry. Prognostic factors related to maintaining TFR at 2 years were age, duration of prior dasatinib, and prior therapy line.

A pre-specified interim analysis of EURO-SKI, which assessed TKI discontinuation in a prospective, non-randomized trial of 755 CML patients who had received TKI for ≥3 years without treatment failure (according to ELN recommendations) and with confirmed DMR for at least 1 year, concluded that patients who achieved long-term DMR have good MRFS and should be considered for TKI discontinuation (31).

In a retrospective analysis of 293 Italian patients with CP-CML in DMR who discontinued therapy with various TKIs (38), patients discontinuing second-generation TKIs had a median duration of treatment with the last TKI of 50 vs. 96 months of treatment with imatinib. Furthermore, using multivariate Cox regression model, a better probability of TFR for patients treated with second-generation TKIs, with an estimated 57% relative risk reduction vs. imatinib, was observed.

In a Spanish retrospective study of 236 CP-CML patients who discontinued TKI treatment outside of clinical trials between April 2009 and February 2018 (patients had received TKI for ≥3 years with sustained MR^4.5^ in ≥4 consecutive assessments over a minimum of 2 years prior to discontinuation) (56), treatment-free survival at 4 years was 64%; there were no cases of disease progression and, in patients who failed to maintain TFR, a DMR was regained 3–5 months after treatment was restarted. A shorter duration of both TKI treatment (<5 years) and time in MR^4.5^ prior to discontinuation (<4 years) were shown by univariate analysis to significantly increase the risk of molecular relapse.

To date, nilotinib is the only TKI with TFR included in the Summary of Product Characteristics (3). According to the updated dosing information, patients taking nilotinib for ≥3 years who are in sustained molecular remission (MR^4.5^, *BCR-ABL* transcripts of ≤0.0032%) and can undergo appropriate monitoring may be eligible to discontinue nilotinib. Implications for clinical practice have recently been reviewed by Pulte and colleagues (57).

### Potential Issues of Treatment-Free Remission

TKI withdrawal syndrome (TKI WS), which presents as musculoskeletal pain, has been reported in patients shortly after stopping TKIs (58). For example, in the ENESTfreedom study, 34% of patients reported musculoskeletal pain-related events during the 1st year of the TFR phase, decreasing to 9, 3, and 3% during the 2nd, 3rd, and 4th year (59). In a systematic review and meta-analysis on the efficacy and safety of TKI discontinuation (10 studies, *n* = 1,601), the weighted mean incidence of TKI WS was 27%, with TKI WS appearing during the early TFR phase (60).

Longer duration of TKI treatment and history of osteoarticular symptoms were identified as risk factors that predispose patients to TKI WS in an analysis of 427 CML patients from the combined cohort of STIM2 and EURO-SKI trials (61). TKI WS, which mainly involved the upper body joints, occurred in 23% of all patients (i.e., 20.4, 41.4, or 40% of patients developed TKI WS after stopping imatinib, nilotinib, or dasatinib, respectively), and WS was confirmed to be a TKI class effect. Therefore, one might speculate that musculoskeletal pain is the result of rebound of symptoms previously suppressed by TKIs. The optimal management of TKI WS is still poorly defined but based mainly on non-steroidal anti-inflammatory drugs.

Cognitive dysfunction has also been reported following the discontinuation of TKIs in patients with CML, highlighting the necessity for vigilance regarding any unexplained neurological occurrences (62).

The potential occurrence of sudden blast crisis should also be mentioned: recently, a case was reported in the STOP-2-Generation TKIs trial (63) and in a CP-CML patient who attempted dasatinib discontinuation under clinical trial conditions (NCT00254423) (64). These rare events required further biological evaluation and as well as prospective assessment in real-life, although this possibility seems to be minimal based on data available from a number of discontinuation trials to date.

## Patient Management Strategies Aimed to Improve the Way to Treatment-free Remission

### The Role of Communication and Adherence

Clinicians' attitude toward treatment discontinuation has changed over time. Nowadays, discontinuation of TKI treatment is considered to be a safe procedure, whereas up until a few years ago patients were more reluctant. Better knowledge of patient motivations and concerns is imperative to facilitate TFR as part of CML management (65). Results from a survey of 87 CML patients eligible for TFR showed that 81% were willing to attempt TFR, with the most common motivations being TKI toxicity (*n* = 26) and inconvenience (*n* = 18). However, the fear of consequences of stopping TKI was the leading reason for reluctance (*n* = 16), and was associated with an incomplete understanding of current data and the need for further information. This survey showed that most patients in DMR would be willing to attempt TFR if recommended by their treating physician and they were provided information on the risk/benefits of TKI discontinuation as well as their likelihood of maintaining TFR (65). In addition, a multicenter survey on patient attitudes toward TFR in 329 CML patients identified side effects (56%), high cost (52%), inconvenience (42%), and pregnancy need (41%) as the main reasons for preferring TFR; younger patients with shorter duration of disease and higher burden of disease symptoms were more likely to attempt TFR (66).

Perceptions about future discontinuation of TKIs were also assessed in a patient-based survey of 1,133 CP-CML patients (67). Overall, 42% of patients had postponed a dose on occasion and therapy had been discontinued by 58% of patients, which was mainly due to forgetfulness or side effects. Approximately half of the patients surveyed were unwilling to discontinue TKI treatment due to the fear of losing the beneficial results they had achieved. Together, these results suggest the need for communication improvement regarding the importance of DMR achievement over time as predisposition to future potential TFR.

The European Steering Group offers a patient-centered approach that educates patients about their treatment options, including TFR, to facilitate patient-physician relationship and to meet the needs of patients (emotional and psychological) within the CML community (68). It clearly sets out recommendations, based on a summary of the Steering Group discussion, for physicians and CML patients considering TKI therapy discontinuation. It outlines key topic areas for patient-physician consideration regarding discontinuation and implications of attempting TFR, as follows: CML treatment goals; what is TFR and when is it appropriate; which patients might and might not be eligible to attempt TFR; patient considerations for discontinuing TKI; TKI WS; psychological implications; molecular recurrence and retreatment.

Moreover, an Italian sub-study of ENESTPath examined the psychological and emotional characteristics of CML patients who failed TFR, defined as either ineligibility for TKI discontinuation due to unstable molecular response (*n* = 8) or relapse during TFR necessitating the reintroduction of nilotinib (*n* = 6) (69). A slightly negative emotional experience was reflected in patients' perception of TFR failure highlighting the emotional distress experienced by CML patients. Patients were also reluctant to believe that TFR success could lead to healing, which is not unfounded since BCR-ABL DNA remains detectable even in patients who have a successful TFR.

Poor adherence to therapy was shown to significantly reduce molecular response rates in 87 patients with CML-CP treated with imatinib (70). Several factors associated with high adherence to TKI therapy have been reported including older age, male sex, management of side effects, daily administration, and patients feeling well-informed about CML by their doctor (71). Importantly, a recent Polish study (72) reported there to be no differences in treatment adherence in CML patients treated with imatinib, dasatinib or nilotinib.

An optimal adherence may have an impact on TFR probability, and should be recalled as a key factor among strategies for maximizing the chance of achieving DMR. Risk factors for a lower adherence should be identified and addressed in order to increase the possibility of achieving stable DMR with a shorter treatment. This is even more important considering that previous reports indicate a higher risk of non-adherence in younger patients, for whom treatment discontinuation is even more important in the long-term to avoid possible specific toxicities (71).

### Molecular Monitoring

Prior to TKI discontinuation, a minimum of 3 years of approved TKI therapy is recommended, with prior evidence of quantifiable *BCR-ABL1* transcripts and ≥2 years of stable molecular response (MR^4^; *BCR-ABL1* 0.01% IS) documented by ≥4 tests performed ≥3 months apart (29). Molecular monitoring post-treatment suspension is also imperative for CML patients attempting TFR (Table 1) but the minimum safe frequency is unknown. Recently, a comparison of current NCCN recommendations (monthly qPCR monitoring of *BCR-ABL1* for 12 months following TKI discontinuation) with less frequent monitoring (monthly testing for 6 months followed by monitoring every 2 months; monitoring every 2 months for 6 months followed by monitoring every 3 months; monitoring every 3 months) was retrospectively applied to data from 107 patients who attempted TFR (73). Monitoring every 2 months for 6 months followed by monitoring every 3 months provided the optimal balance between reduced monitoring and minimized the delay in detecting relapse and recommencement of TKI. With reduced monitoring frequency, a rapid turnaround time for PCR results is imperative as the retrospective analysis also identified substantial increases in *BCR-ABL1* transcripts between detection of molecular relapse and TKI recommencement (73).

A prerequisite for TKI discontinuation is standardized molecular monitoring (8). The EUREKA registry evaluated the assessment of DMR in CP-CML patients using standardized molecular monitoring collected in the real-life practice context, and compared results with EUTOS-certified laboratories (74). Results showed substantial agreement between EUTOS-certified and local laboratories demonstrating reliable molecular monitoring outside of clinical trials across six European countries. In Italy since 2008, LabNet supports the diagnostics of CML in 55 laboratories through standardization, collaboration, and quality controls.

## Future Directions in Treatment and Monitoring to Improve TFR Outcomes

### New Drugs and Novel Strategies

Asciminib binds to the myristoyl pocket of ABL1 and is a potent and specific inhibitor of BCR-ABL1. Data from preclinical studies demonstrated cooperativity when asciminib was combined with TKIs, suggesting that this combination may provide more potent inhibition of BCR-ABL1 and prevent the emergence of resistance mutations. Asciminib demonstrated clinical activity and good safety and tolerability as monotherapy in heavily pretreated CML patients with resistance or intolerance to at least two prior TKIs in an ongoing phase 1 study (NCT02081378) (75). Preliminary results from the same trial have also demonstrated a good safety profile and promising efficacy for the combination of asciminib plus imatinib in 25 CP-CML patients (76), and for asciminib plus nilotinib or dasatinib in 34 CML patients in chronic or accelerated phase (77). Promising results of asciminib monotherapy in heavily pretreated patients were published recently (75). An ongoing phase 2 clinical trial (clinicaltrials.gov identifier: NCT03578367) is investigating asciminib plus imatinib in patients not achieving a DMR with imatinib, with an estimated primary completion date of March 2021.

The rationale for using IFN-α in order to maintain/improve molecular response after TKI discontinuation was initially demonstrated by Burchert et al. (78). Indeed, the combination of IFN-α plus TKI has demonstrated good molecular responses in CML patients, with significantly higher rates of molecular response identified in patients treated with imatinib plus IFN-α or with PEGylated IFN (Peg-IFN) alpha-2a compared with imatinib alone (79, 80). Similarly, dasatinib plus Peg-IFN alpha-2b showed promising efficacy in a phase 2 trial of 40 CP-CML patients (81), and Peg-IFN alpha-2a combined with nilotinib resulted in DMR, albeit with substantial toxic effects, in a phase 2 study of 41 CML patients, with 7 (17%) patients achieving MR^4.5^ after 1 year of treatment (82). The ongoing TIGER trial (NCT01657604; estimated primary completion date December 2020) aims to assess the efficacy and safety of nilotinib compared with nilotinib plus Peg-IFN alpha-2b as first-line therapy in newly diagnosed CP-CML patients who are Ph/BCR-ABL positive.

An interim analysis of the PETALS study, which evaluated cumulative rates of MR^4.5^ following 12 months of treatment with nilotinib or nilotinib plus IFN in 200 newly diagnosed CP-CML patients, showed statistically significant DMR rates in favor of the combination treatment arm at 12 months (83). Similarly, interim results of the PINNACLE study also suggested that combination therapy with nilotinib plus Peg-IFN alpha-2b results in favorable rates of molecular responses compared with nilotinib as monotherapy (84). TFR rate data on IFN-TKI combinations are still unknown.

Other agents are being evaluated in clinical studies, including HQP1351 (85) and PF-114 (86), however results to date suggest that safety may be an issue.

Other promising combinations and ongoing trials in CML patients include ruxolitinib in combination with nilotinib (87) or in combination with a TKI (NCT03610971), venetoclax in combination with dasatinib (NCT02689440), pioglitazone (a peroxisome proliferator-activated receptor γ agonist) in combination with imatinib (88) or in combination with a TKI (NCT02889003), dipeptidyl peptidase IV inhibitors (89), which are also under investigation in the European clinical trial CAMN107CNL08T (EudraCT Number: 2017-000899-28), and the ACTIW study (NCT02767063).

A de-escalation approach to TFR is among the novel strategies intended to improve TFR outcomes. Dose modification of TKI therapy aims to reduce adverse events in CML patients whilst maintaining efficacy. Indeed, a recent review concluded that dose optimization with adequate monitoring was feasible and safe, and improved quality of life (90).

The effect of gradual TKI withdrawal prior to discontinuation was investigated in the non-randomized, phase 2 De-Escalation and Stopping Treatment with Imatinib, Nilotinib, or Dasatinib (DESTINY) study (91). At study entry, 148 patients had been treated with imatinib, 16 with nilotinib, and 10 with dasatinib for a median duration of 6.9 years; all patients had at least a 1-year stable MMR. Patients were assigned to the MR^4^ group if all PCR measurements during the observation period were ≤0.01%, and to the MMR group if any PCR measurements were >0.01% but ≤0.1%. TKI treatment was de-escalated to half the standard dose for 12 months, then stopped for 24 months. Of the 125 patients in the MR^4^ group, 84 (67%) achieved the primary end-point (no loss of MMR at 36 months), with a 3-years MRFS of 72% (95% CI 64–80). Similarly, 16 of 49 (33%) patients in the MMR group completed the study, with a 3-years MRFS of 36% (95% CI 25–53). Overall, this study suggests that an initial TKI de-escalation period prior to TFR in patients with stable MR^4^ might improve the TFR rate, with a lower incidence of WS. The phase II, prospective multicenter De-escAlation and discontinuation of Nilotinib ThErapy (DANTE) trial is currently ongoing in Italy to further elucidate this scenario.

### Enhanced Detection Methods

Improved accuracy in detecting *BCR-ABL1* transcripts supports the selection of CML patients eligible for TFR, and the emergence of digital PCR (dPCR) may offer a more sensitive and accurate detection than real-time quantitative PCR (qPCR). The reliability and efficiency of dPCR was evaluated in 142 CML patients treated with TKIs, assessing ≥2 years DMR using real-time qPCR (92). dPCR detected measurable transcripts that were undetectable by conventional real-time qPCR, and identified differing quantities of *BCR-ABL1* transcripts in patients within the same MR class. For the detection and monitoring of *BCR-ABL1* molecular levels, digital PCR was more accurate and sensitive compared with real-time qPCR, highlighting its potential to improve stable DMR recognition and hence the selection of patients eligible for TKI discontinuation (92).

Enhanced detection methods also include digital droplet PCR, which was found to be predictive of TFR outcomes when tested at the time of TKI discontinuation in CP-CML patients (93). Notably, residual leukemic cell load determined by digital droplet PCR was one of two key factors for predicting a successful TFR in CP-CML patients (94).

The development of more sensitive detection methods is not separate from the optimization of known methods. Monitoring *BCR-ABL1* using the newly implemented TaqMan system with *GUSB* as reference gene (TM/*GUSB*) was compared with the LightCycler quantitative reverse transcription (qRT)-PCR system, which utilizes *ABL1* as reference gene (LC/*ABL1*) (95). Overall, TM/*GUSB* was found to be a robust and reliable method for monitoring CML patients, showing improved sensitivity compared with LC/*ABL1*. The enhanced sensitivity of TM/*GUSB* may support in loss of response prediction.

Highly sensitive individualized BCR-ABL1 DNA PCR was used to show gradually reducing levels of residual CML cells in long-term TFR CML patients, with a continuous rate of decline in *BCR-ABL1* transcripts up to 3 years after imatinib discontinuation demonstrated in nine patients in long-term stable TFR (96). The likelihood of residual BCR-ABL1 in TFR patients being due to the presence of BCR-ABL1 in clonal lymphocytes (46), which may be reducing over time (96), needs to be clarified.

### Second Attempt at TKI Discontinuation

Whether patients who relapse on TFR should re-attempt TKI discontinuation is currently unknown. The RE-STIM study, which evaluated TFR in 70 CML patients who underwent a second TKI discontinuation after a failed first attempt, demonstrated that a first unsuccessful attempt at TKI discontinuation did not prevent success during a second attempt (97). In total, two thirds of patients (64%) experienced MMR loss after a median of 5.3 months off therapy. TFR rates were 48, 42, and 35% at 12, 24, and 36 months, respectively. Univariate analysis identified the speed of molecular relapse after the first TKI discontinuation attempt as being the only factor significantly associated with outcome during the second attempt; the TFR probability at 24 months was significantly higher in patients who remained in DMR at 3 months after the first attempt to discontinue TKI compared with those who lost MMR within 3 months (72 vs. 36%). A more recent analysis of 106 CP-CML patients with 41 months of follow-up from the RE-STIM study confirmed the safety and success of a second TKI discontinuation attempt (98). Major factors significantly associated with TFR outcome were the speed of molecular relapse after the first TKI discontinuation and TKI-free duration of >6 months after the first attempt at TKI discontinuation.

Studies assessing a second TKI discontinuation attempt after a failed first attempt and re-treatment with nilotinib (NCT02917720), dasatinib (NCT03573596) or ponatinib (NCT04043676) are currently ongoing.

## Conclusions

The number and potency of available treatments for CML patients have significantly increased in recent years, making therapeutic decisions more complex and treatment goals more ambitious. In this scenario the optimal evaluation and management of patients' comorbidities is needed to allow an individualized treatment without reducing treatment options. DMR and TFR are feasible treatment goals in CML and criteria for TKI discontinuation in clinical practice have been published based on expert recommendations and formal guidelines. Optimizing treatment to reach DMR is imperative and studies have shown that the use of second-generation TKIs, and in particular nilotinib, may increase the proportion of potential candidates eligible for TKI discontinuation through the reach of faster and deeper responses, although patient selection is very important (e.g., patients at high cardiovascular risk should be excluded). Patient management strategies aimed at improving TFR are essential and include communication to facilitate the patient-physician relationship and to meet the needs of patients (emotional and psychological). New drugs (i.e., asciminib) and treatment strategies (i.e., IFN-α combined with a TKI and de-escalation prior to TKI discontinuation), as well as novel molecular monitoring strategies (i.e., digital PCR) and treatment regimens (i.e., repeat attempts at TKI discontinuation) aimed toward improving TFR outcomes are currently under evaluation and may improve TFR prediction and outcomes.

Although TFR success rate is similar when stopping first- or second-generation TKIs, second-generation TKIs lead to more rapid and deeper molecular responses. Hence, the achievement of TFR is becoming increasingly achievable in CML patients; however in the near future we may further improve the path to TFR and widen the TFR population in clinical practice.

## Author Contributions

MA, MBo, MBr, FC, AG, AI, PP, FS, and GS contributed to manuscript revision, read and approved the submitted version.

## Conflict of Interest

MA has received honoraria from Novartis and Incyte. MBo has received research funding from Novartis, honoraria from Bristol-Myers Squibb, Pfizer, Incyte, and Amgen. MBr, AI, and PP have received honoraria from Novartis, Bristol-Myers Squibb, Pfizer, and Incyte. FC has consulted for and received honoraria from Novartis, Incyte, Pfizer, and Bristol-Myers Squibb. FS has received honoraria from Bristol-MyersSquibb, Incyte, Novartis, and Pfizer. The remaining authors declare that the research was conducted in the absence of any commercial or financial relationships that could be construed as a potential conflict of interest.
